# Supplementary figures and images for: Rescue of bmp15 deficiency in zebrafish by mutation of inha reveals mechanisms of BMP15 regulation of folliculogenesis
Source: PLoS Genet. 2023 Sep 15;19(9):e1010954. doi: 10.1371/journal.pgen.1010954 (PMC10529593; doi:10.1371/journal.pgen.1010954)

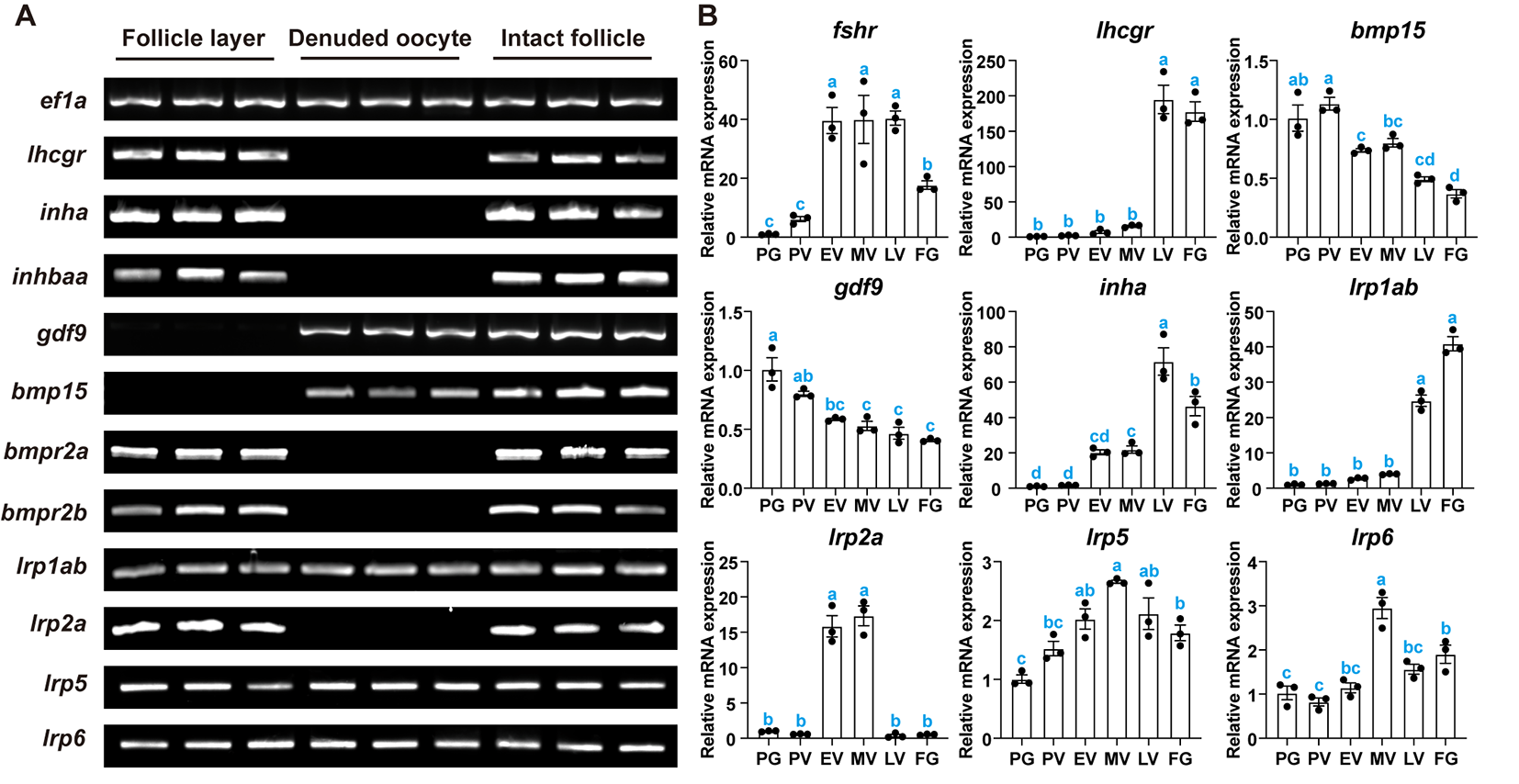

Supplement: S1 Fig — (A) Spatial distribution of gene expression in the FG follicle. The housekeeping gene ef1a was expressed in both oocyte and follicle layer, whereas the marker genes lhcgr and gdf9 were expressed in the follicle layer and denuded oocyte respectively, indicating clean separation of the two compartments. The expression of bmp15 was detected exclusively in the denuded oocyte, whereas inha, inhbaa and two BMP type II receptors (bmpr2a and bmpr2b) were detected only in the follicle layers. In addition, lrp1ab, lrp5 and lrp6 were all expressed in both follicle layer and oocyte whereas lrp2a was exclusively expressed in the follicle layer. (B) Temporal expression profiles of bmp15 and other genes during folliculogenesis. The expression patterns of fshr and lhcgr were used as the internal reference for appropriate staging of the follicles. The relative mRNA levels were determined by real-time qPCR, normalized to ef1a, and expressed as fold change compared with the levels at the PG stage. The values are the mean ± SEM (n = 3 fish) from a representative experiment and analyzed by ANOVA followed by the Tukey HSD for multiple comparisons. Different letters indicate statistical significance (P < 0.05). PG, primary growth; PV, previtellogenic; EV, early vitellogenic; MV, mid-vitellogenic; LV, late vitellogenic; FG, full-grown. (TIF) [file pgen.1010954.s001.tif]

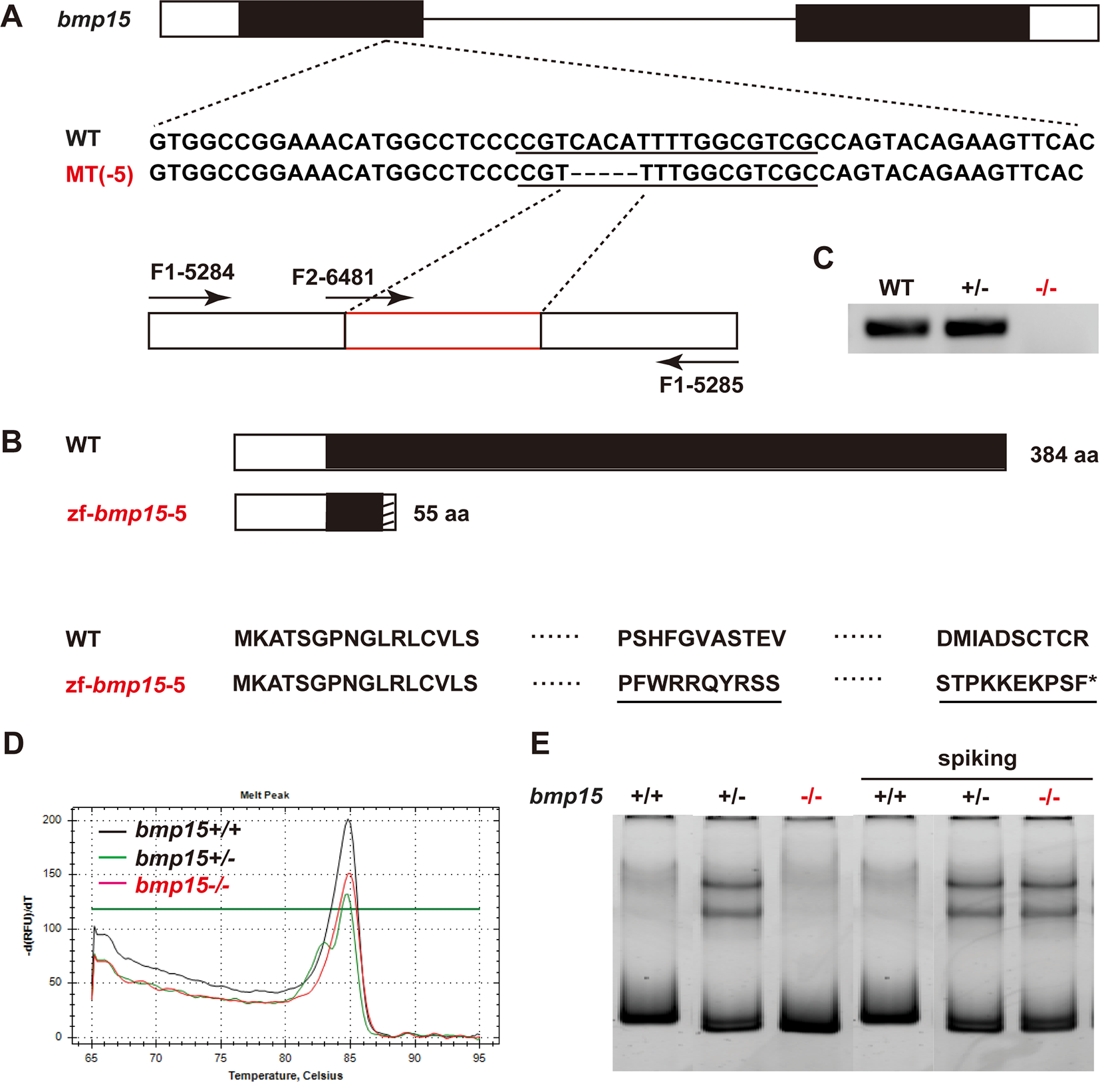

Supplement: S2 Fig — (A) Schematic illustration of the genomic structure of zebrafish bmp15 gene. The black and white boxes indicate the coding and non-coding regions of the exon, respectively. The underlined sequence indicates CRISPR/Cas9 target site, and the dashed line indicates the deleted sequence (-5 bp) of zebrafish bmp15. The primers for mutation screening (bmp15_5284_F/bmp15_5285_R) are shown below. (B) Schematic representation of Bmp15 amino acid (aa) sequence. The 5-bp deletion introduced a premature termination codon, resulting in the synthesis of a truncated protein of 55 aa. (C) Confirmation of mutation at the mRNA level in the ovary. RT-PCR was performed on total RNA extracted from WT, heterozygous and homozygous mutant ovaries with a specific primer (bmp15_6481_F/bmp15_5285_R) overlapping with the deleted sequence. No signal could be detected in the mutant ovary. (D) Genotyping with HRMA. Melt curves for WT, heterozygotes and homozygous mutant are marked in black, green, and red respectively. (E) Genotyping with HMA. The heterozygotes showed two additional bands and the homozygous mutant showed a smaller band than WT. (TIF) [file pgen.1010954.s002.tif]

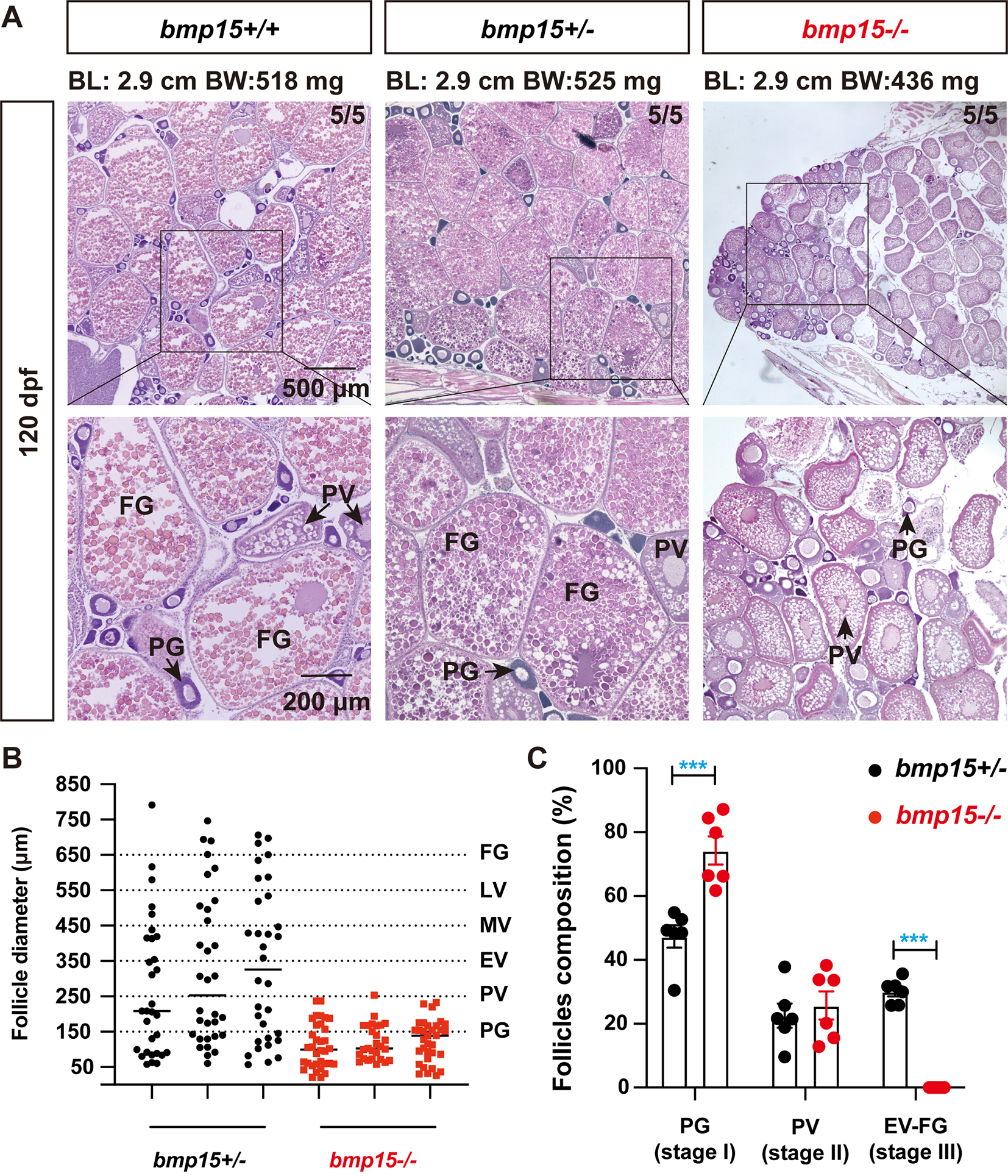

Supplement: S3 Fig — (A) Histology analysis of the control (bmp15+/-) and mutant (bmp15-/-) fish at 120 dpf. Follicle development was arrested at PV stage in bmp15-/- mutant. (B) Size distribution of follicles in the control (bmp15+/-) and mutant (bmp15-/-) fish at 120 dpf (n = 3). The mutant follicles could grow to the size of PV stage only (~250 μm), while the control follicles could reach FG stage (> 650 μm). (C) Follicle composition analysis at 120 dpf. Compared with the control, the bmp15-/- ovary contained significantly more PG (stage I) follicles but no vitellogenic follicles (EV–FG, stage III). The values are expressed as mean ± SEM (n = 6) and analyzed by ANOVA followed by the Tukey HSD for multiple comparisons (*** P < 0.001). (TIF) [file pgen.1010954.s003.tif]

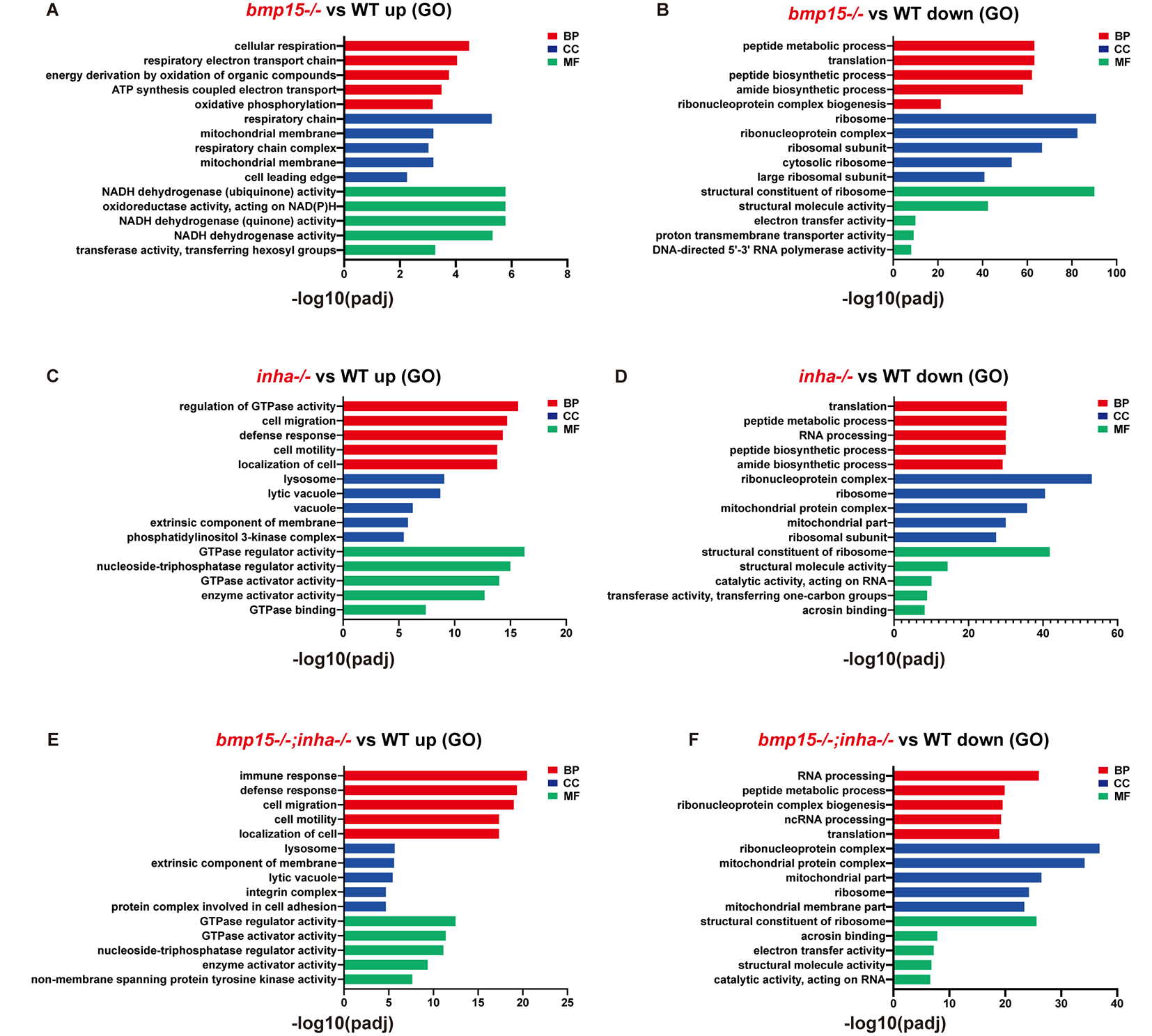

Supplement: S4 Fig — PG follicles from the wild type (WT) control (bmp15+/+;inha+/+) and mutants (bmp15-/-, inha-/- and bmp15-/-;inha-/-) were used for RNA-seq analysis. GO terms include three parts: biological process (BP), cellular component (CC) and molecular function (MF). (TIF) [file pgen.1010954.s004.tif]

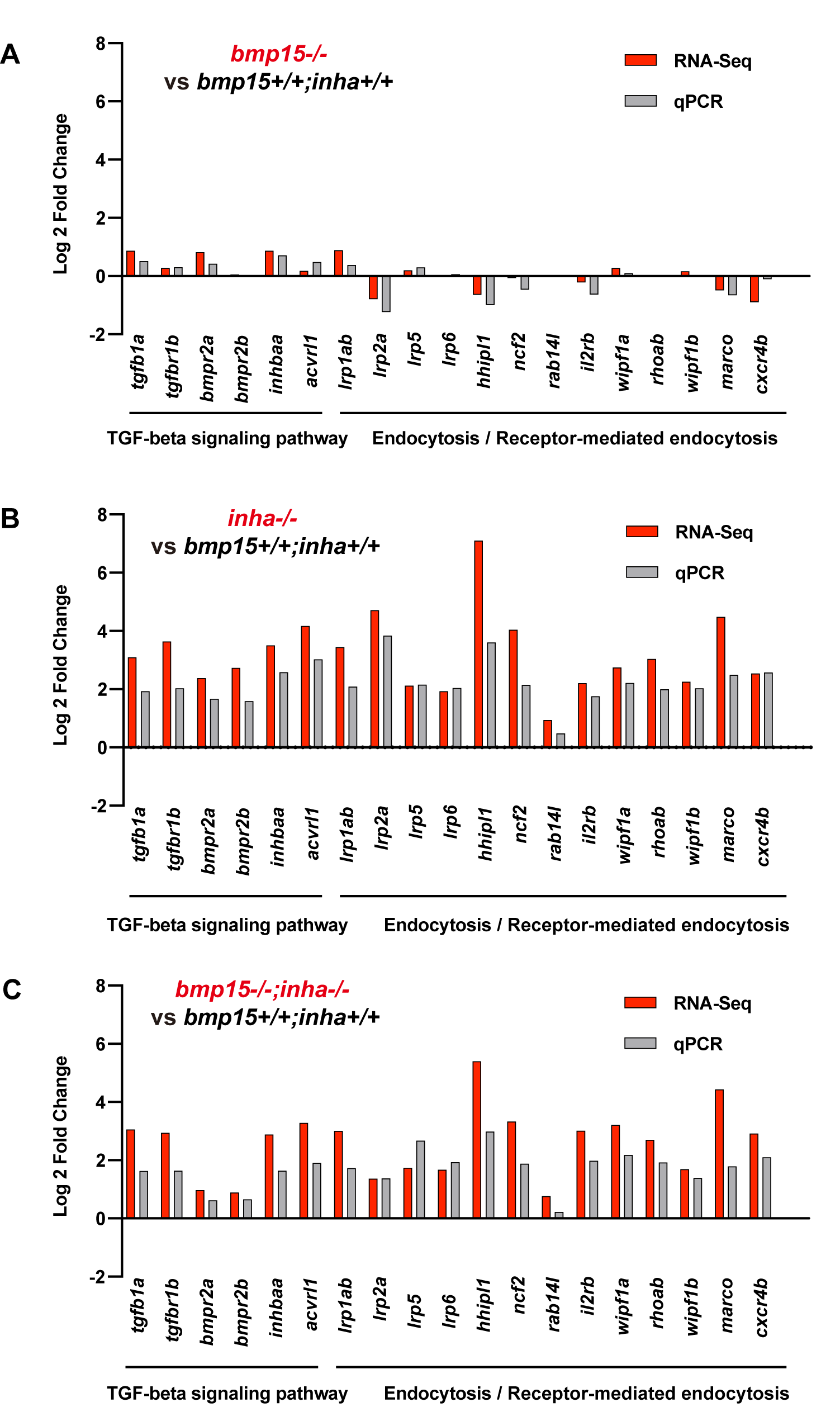

Supplement: S5 Fig — The DEGs were identified by RNA-seq analysis and confirmed by RT-qPCR. (TIF) [file pgen.1010954.s005.tif]

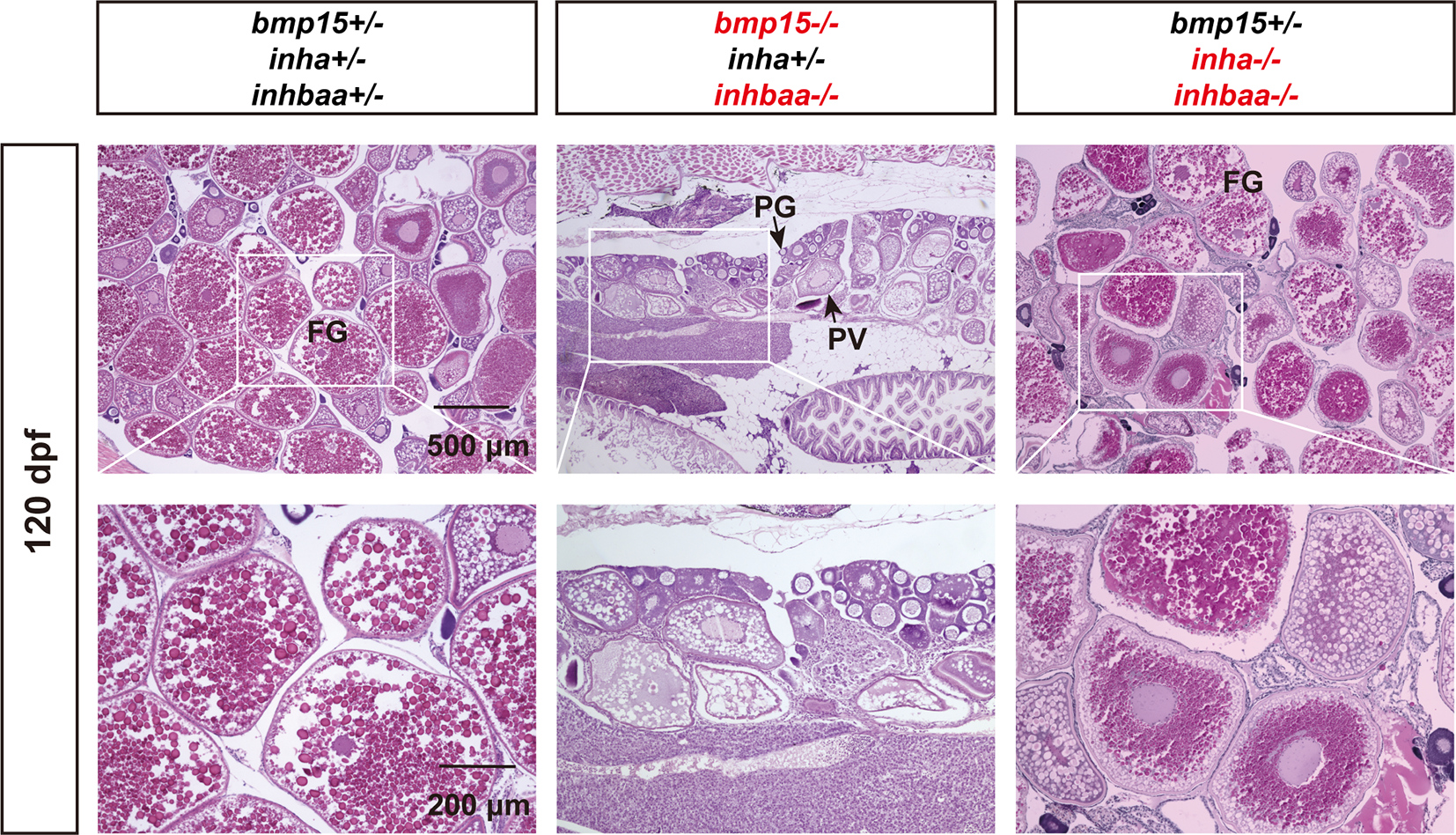

Supplement: S6 Fig — The follicle development was arrested at PV stage in bmp15-/-;inhbaa-/- double mutant while the follicles showed normal vitellogenic growth in inha-/-;inhbaa-/- double mutant. PG, primary growth; PV, pre-vitellogenic; FG, full-grown. (TIF) [file pgen.1010954.s006.tif]

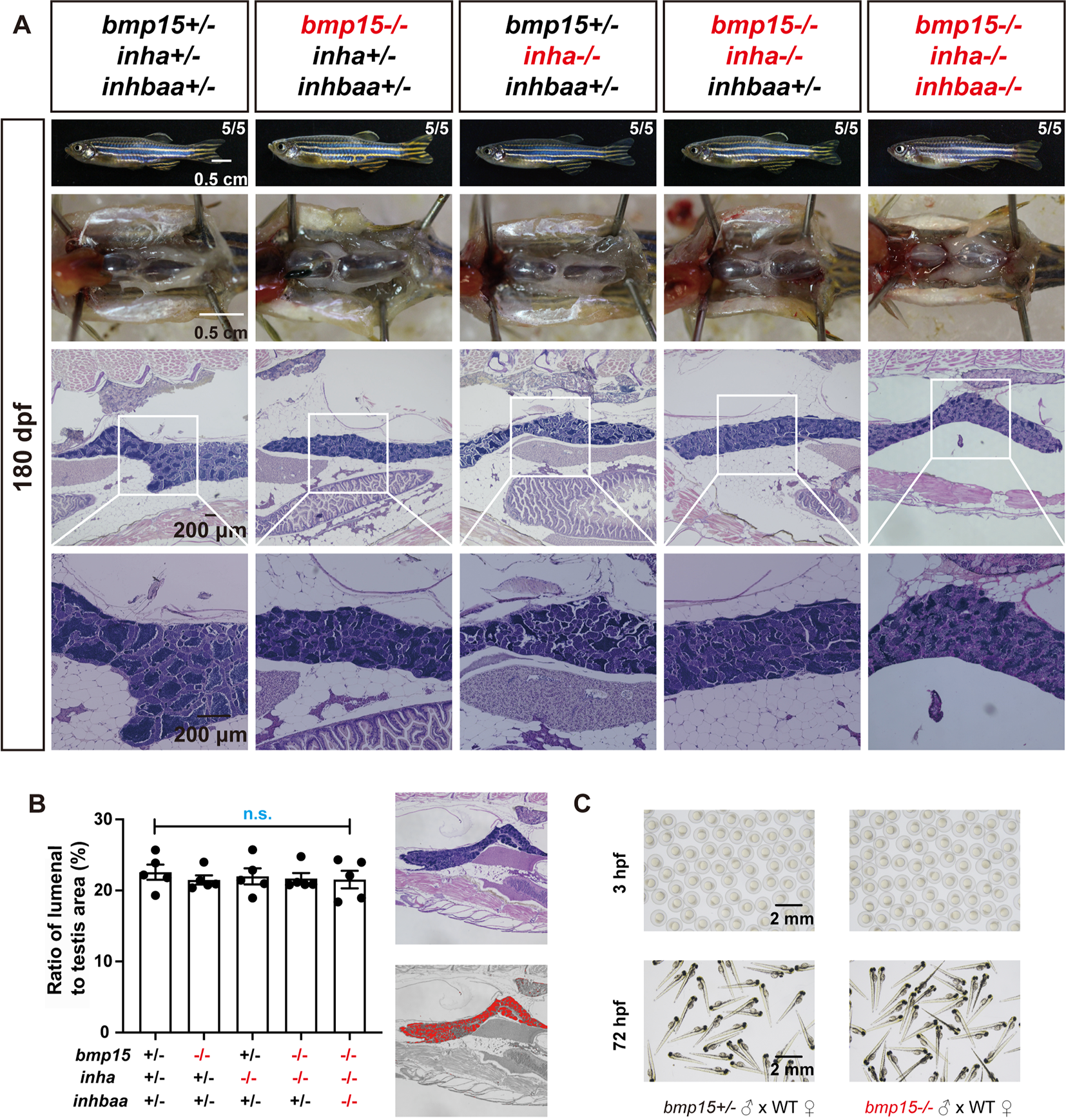

Supplement: S7 Fig — (A) Morphology, gross anatomy, and histological analysis of sexually mature males at 180 dpf. The boxed areas are shown at higher magnification below. (B) Area of the spermatozoa-filled luminal spaces in the testis. There was no significant difference among these mutants compared with the control at 180 dpf. The values are expressed as mean ± SEM (n = 5) and analyzed by ANOVA followed by the Tukey HSD for multiple comparisons. (C) Offspring of control (bmp15+/–) and mutant (bmp15–/–) males with WT females (bmp15+/+) at 3 hpf and 72 hpf. (TIF) [file pgen.1010954.s007.tif]
