# Supplementary material for: Rescue of bmp15 deficiency in zebrafish by mutation of inha reveals mechanisms of BMP15 regulation of folliculogenesis
Source: PLoS Genet. 2023 Sep 15;19(9):e1010954. doi: 10.1371/journal.pgen.1010954 (PMC10529593; doi:10.1371/journal.pgen.1010954)
Supplement: S4 Table — (DOCX) [file pgen.1010954.s011.docx]

**Table S1. Primer used for CRISPR, HRMA and RT-PCR**

| Gene | Primer ID | Primer Sequence | Application |
| --- | --- | --- | --- |
| *bmp15* | 4130 | TAGGCGACGCCAAAATGTGACG | CRISPR |
| *bmp15* | 4131 | AAACCGTCACATTTTGGCGTCG | CRISPR |
| *bmp15* | 5284 | ACTCTGCGTACTGTCCTGTTT | HRMA |
| *bmp15* | 5285 | ACGGGAGGTCTAAAATGAGGGT | HRMA |
| *gdf9* | 1670 | ATTATGGCGACGCTGTTTTT | HRMA |
| *gdf9* | 1671 | CGTTTTCAAAGTTGTAGCTTGATG | HRMA |
| *inha* | 1676 | TTTTCTCCTCCATCGGTTCA | HRMA |
| *inha* | 1677 | CATCCAACCCCAAACCTTC | HRMA |
| *inhbaa* | 4136 | ATGTCCCCTCTGCCTCTACT | HRMA |
| *inhbaa* | 4137 | AGGCTCCCTTTGGTGACCAT | HRMA |
| *fshb* | 5836 | CAGATGAGGATGCGTGTGC | RT-PCR |
| *fshb* | 5837 | ACCCCTGCAGGACAGCC | RT-PCR |
| *lhb* | 983 | ATGTTATTGGCTGGAAATGG | RT-PCR |
| *lhb* | 984 | CTAGTATGCGGGGAAATCC | RT-PCR |
| *fshr* | 957 | AACATGCACATAGAGAGGATTCCCAG | RT-PCR |
| *fshr* | 958 | GCTCAGTAAACAGCTCCAGGC | RT-PCR |
| *lhcgr* | 954 | TGAATACGCCACAATGAATCTCTT | RT-PCR |
| *lhcgr* | 955 | ATGACGATCCAATGACATCTGACTC | RT-PCR |
| *ef1a* | 728 | GGCTGACTGTGCTGTGCTGATTG | RT-PCR |
| *ef1a* | 729 | CTTGTCGGTGGGACGGCTAGG | RT-PCR |
| *cyp19a1a* | 818 | TGTGCGTGTCTGGATCAATGG | RT-PCR |
| *cyp19a1a* | 819 | AAGCCCTGGACCTGTGAGAG | RT-PCR |
| *esr1* | 1220 | GTCTCAAAGCCATCATACTCATCAATTC | RT-PCR |
| *esr1* | 1221 | TTCATTCGGTATAAGTGCTCCATTCC | RT-PCR |
| *esr2a* | 1226 | CGACTTCAACAGAACCATGCTACTAG | RT-PCR |
| *esr2a* | 1227 | CTTCACACGACCACACTCCATAATG | RT-PCR |
| *esr2b* | 1230 | CAGTCCCTCTCAGCACCTCTTTC | RT-PCR |
| *esr2b* | 1231 | TATCCAGCCAGCAGCATTCCAG | RT-PCR |
| *vtg1* | 4009 | CTGCGTGAAGTTGTCATGCT | RT-PCR |
| *vtg1* | 4010 | GACCAGCATTGCCCATAACT | RT-PCR |
| *vtg2* | 6998 | GGACTGGCCAAAGCAGGTAT | RT-PCR |
| *vtg2* | 6999 | CCAAGTGCCAGCATACTCGT | RT-PCR |
| *vtg3* | 4393 | AACTGCCACACCTGGTTGAA | RT-PCR |
| *vtg3* | 4394 | TGATCTCGGCAGACAGATGC | RT-PCR |
| *vtg4* | 7000 | TCAGTGCCGTGACTGAGAAC | RT-PCR |
| *vtg4* | 7001 | GATCTGAAGCTGAGCAGCCA | RT-PCR |
| *vtg5* | 7002 | GCTCTGCTTTTGGGAGGTCT | RT-PCR |
| *vtg5* | 7003 | CCAAGTGCCAGCATACTCGT | RT-PCR |
| *vtg7* | 7004 | CAGCAGCAAGGTTCTCCTCA | RT-PCR |
| *vtg7* | 7005 | CCAAGTGCCAGCATACTCGT | RT-PCR |
| *lrp1ab* | 7006 | TGTCCAGACGGCTCTGATGA | RT-PCR |
| *lrp1ab* | 7007 | CACATCCGTTCCTCGACACT | RT-PCR |
| *lrp2a* | 7008 | GGATTTTTCCGTTGCGGGAC | RT-PCR |
| *lrp2a* | 7009 | TCAAGCACTGGAACTGAGCG | RT-PCR |
| *lrp5* | 7010 | GAGCGCTTGCGATGGAGATT | RT-PCR |
| *lrp5* | 7011 | ACTGCAGACTCTGAGCGACC | RT-PCR |
| *lrp6* | 7012 | GTGGAAGGATCTGGACAGCC | RT-PCR |
| *lrp6* | 7013 | CATCGCTGCACGGTCTATCT | RT-PCR |
| *gdf9* | 206 | GAGTCTGTTGAACCCGACG | RT-PCR |
| *gdf9* | 207 | GCAGGTGGATGTCCTTCTTA | RT-PCR |
| *inha* | 520 | AGCCTCCTCTGCCAGTGTTG | RT-PCR |
| *inha* | 521 | ATGTTGATGGAAGCGATGGTCTC | RT-PCR |
| *inhbaa* | 929 | GACCGAACAGGCAGAACAG | RT-PCR |
| *inhbaa* | 930 | GTCCACCACAGACATCTCACC | RT-PCR |
| *inhbab* | 1063 | ACGGCACAGTGGAGATGG | RT-PCR |
| *inhbab* | 1064 | CAGGACATCAGGGGCATC | RT-PCR |
| *inhbb* | 6109 | GCGGGTAAAGTTAGGGAG | RT-PCR |
| *inhbb* | 6110 | AGAGGCTGGACTTGGATG | RT-PCR |
| *fsta* | 6101 | TTTTATTACTCTTTTGGCTCTG | RT-PCR |
| *fsta* | 6102 | CATTCCTCCCGACTCATC | RT-PCR |
| *fstb* | 6103 | ACTTGATGGAGGAGCAGAA | RT-PCR |
| *fstb* | 6104 | GTACCCAAACGACCACTTT | RT-PCR |
| *vtg6* | 3991 | AAGTCAGCAGCAAGGTTCGT | RT-PCR |
| *vtg6* | 3992 | AGGTGAGCTTAGTGGCAGGA | RT-PCR |
| *bmp15* | 6300 | TGGGTCCAACACCATAAGACTG | RT-PCR |
| *bmp15* | 6301 | GACGCCTTCACCAGTTTGTC | RT-PCR |
| *bmp15* | 6481 | CATGGCCTCCCCGTCACATT | RT-PCR |
| *bmpr2a* | 4389 | ACCGCCAGCAGTTCACTAATG | RT-PCR |
| *bmpr2a* | 4390 | TCCGTCTTAACCAGCACATTCC | RT-PCR |
| *bmpr2b* | 4391 | GGCTCTGCTCACTGCTTCTG | RT-PCR |
| *bmpr2b* | 4392 | TGCGATGGCGTTGTGGTAAC | RT-PCR |
| *tgfb1a* | 5235 | ﻿GGAATACTGCGCTTCATCTC | RT-PCR |
| *tgfb1a* | 5236 | ﻿GGACTATTTTGGCAAGGAGG | RT-PCR |
| *tgfbr1b* | 5237 | ﻿GAAGATCCAACAGAGGAGGA | RT-PCR |
| *tgfbr1b* | 5238 | ﻿GGTAGAGCACATGATTTGCA | RT-PCR |
| *bmpr2a* | 4389 | ACCGCCAGCAGTTCACTAATG | RT-PCR |
| *bmpr2a* | 4390 | TCCGTCTTAACCAGCACATTCC | RT-PCR |
| *bmpr2b* | 4391 | GGCTCTGCTCACTGCTTCTG | RT-PCR |
| *bmpr2b* | 4392 | TGCGATGGCGTTGTGGTAAC | RT-PCR |
| *acvrl1* | 7370 | ACACCACTGCAATGCAAACC | RT-PCR |
| *acvrl1* | 7371 | GCCATGGCAATCACCAACAA | RT-PCR |
| *hhipl1* | 7374 | TACGTGCAGGATCTGCTGTGT | RT-PCR |
| *hhipl1* | 7375 | TGAGCGCAGTAGTCTGGACA | RT-PCR |
| *ncf2* | 7378 | ACATCGGCTGTCTTTGTCTGAA | RT-PCR |
| *ncf2* | 7379 | GCCAGGTGTTCATCTTTGCCAAT | RT-PCR |
| *rab14l* | 7382 | GCTACTATAGAGGAGCCGCTGG | RT-PCR |
| *rab14l* | 7383 | TGAGGTTTCTGGCGTCCGTC | RT-PCR |
| *il2rb* | 7386 | GCAACGTGTCTTGGAGTAAAGG | RT-PCR |
| *il2rb* | 7387 | GAGAGTTGGCCGGGTTTTGC | RT-PCR |
| *wipf1a* | 7390 | ATGAGAGACCCCCTCCAACA | RT-PCR |
| *wipf1a* | 7391 | GCTCCACCTCTGTTTCCTCC | RT-PCR |
| *rhoab* | 7394 | GTTTCCTGAAGTCTACGTACCA | RT-PCR |
| *rhoab* | 7395 | AGTATCCCACAGAGCGAGTTC | RT-PCR |
| *wipf1a* | 7398 | GGGCAGAAATGCAAGTGGAAGAT | RT-PCR |
| *wipf1a* | 7399 | TCTACACCCGGTCTGTTTGGT | RT-PCR |
| *marco* | 7402 | CTCTGTGAGGCCGAGCGATA | RT-PCR |
| *marco* | 7403 | TGACGTTTGCTCGCTGATTTC | RT-PCR |
| *cxcr4b* | 7406 | TGGCAATGGACTTGTGGTGC | RT-PCR |
| *cxcr4b* | 7407 | ACAAACAGGAGGTCTGCGATT | RT-PCR |
