# Supplementary material for: Rescue of bmp15 deficiency in zebrafish by mutation of inha reveals mechanisms of BMP15 regulation of folliculogenesis
Source: PLoS Genet. 2023 Sep 15;19(9):e1010954. doi: 10.1371/journal.pgen.1010954 (PMC10529593; doi:10.1371/journal.pgen.1010954)
Supplement: Attachment — Submitted filename: Response to reviewers.docx [file pgen.1010954.s012.docx]

**Response to Reviewers’ comments**

We are grateful to the reviewers for their favorable assessment of our work and valuable comments and suggestions, which have significantly improved the manuscript. We have revised the manuscript accordingly and our responses to reviewers’ comments are summarized below.

**Reviewer 1:**

In this study authors investigate the mechanism by which BMP15 regulates zebrafish follicle development. They generated single (*bmp15-/-*), double (*bmp15-/-;inha-/-*) and triple (*bmp15-/-;inha-/-;inhbaa-/-*) mutants to study the interaction of Bmp15 with avidin/inhibin. The evidence provided from genetic and transcriptomic approaches is solid.

However, the new knowledge is embedded in many figures of known information, preventing easy understanding of the paper. The title of the paper also does not deliver clear message about their new findings. Authors should differentiate their new findings from those already known from the literature. For example, it will be nice to simplify their summary figure (Fig. 13) and differentiate their new information from known knowledge. Since many of these mutants have been reported, some known data can be moved to the supplement.

**RESPONSE**

We thank the Reviewer for providing a positive evaluation and constructive feedback on our manuscript. Based on the suggestions by the Reviewer, we have changed the manuscript title to “**Rescue of *bmp15* deficiency in zebrafish by mutation of *inha* reveals mechanisms of BMP15 regulation of folliculogenesis**”. We have also moved some of the data (Fig. 1 and 3) to the supplementary part as they contain some known information, reducing the total number of figures to 11. Some data of known information are included in some other figures because they are mainly used as the reference or control for our new data, making it difficult to separate them.

Regarding the summary figure (Fig. 13, now Fig. 11), another reviewer suggested that we add other factors that have been reported to be also involved in controlling early folliculogenesis and sex reversal. However, that would make the figure even more sophisticated, in contrast to the suggestion by the Reviewer 1. Therefore, we have chosen to maintain the original summary figure with some minor changes to make it clearer (Fig. 11). It would be much appreciated if the Reviewer accepts this.

**Major point 1:**

Authors hope to identify pathways that participate in the rescue of *bmp15* mutants by *inha* mutation using RNA seq in Fig. 6, but the results are not clear. The pathways they identify, eg. TGF-beta and receptor-mediated endocytosis do not seem to be reduced in bmp15 mutants. More detailed statistical comparison will be necessary. Furthermore, these data will need to be validated by real-time qPCR. Further follow-up experiments or at least some explanation will be required to dissect the relationship of bmp15, inha, and ovary development. Please use the same color codes for all samples throughout Fig. 6A-C to facilitate reading. In Fig. 6C, the Y-axis of the normalized read count in the TGF-beta signaling pathway should be normalized and presented as a log2 scale.

**RESPONSE**

We thank the Reviewer for the valuable suggestion. As suggested, we have reanalyzed the data and validated the RNA-Seq results by real-time qPCR during the revision. Since the space was not enough in Fig. 6C, we have moved this part to Fig. S5. The data from RNA-seq and real-time qPCR analyses were both normalized and presented as a log2 scale.

The Reviewer raised an excellent issue regarding no expression reduction of the TGF-beta and receptor-mediated endocytosis pathways in *bmp15-/-* single mutant. This could be due to the stage of follicles used for the RNA-seq analysis. We used PG follicles for the RNA-seq analysis due to the observation that *bmp15-/-* females showed delayed PG-PV transition, suggesting a potential defect in PG follicles. At this stage, many genes were either not expressed or expressed at very low levels, making it difficult to reveal reduced expression. In support of this are the expression of *cyp19a1a* and *inhbaa* and their responses to *bmp15* mutation. They were expressed at low levels at PG stage and showed no response to the mutation of *bmp15*-/-; however, both genes increased their expression significantly at PV stage and the loss of *bmp15* caused a significant decrease of their expression. The loss of *inha-/-* induced a dramatic increase of *cyp19a1a* expression, which could be due to the increased expression of *inhbaa* and therefore production of activin.

We have revised the manuscript substantially on this point. In the Results part, we have added the following: “Interestingly, some genes belonging to the pathways of TGF-β signaling, endocytosis, and receptor-mediated endocytosis showed no change in expression in *bmp15-/-* (Fig. S5A, for details see Supplemental Table 2) but their expression increased significantly in *inha-/-* (Fig. S5B, for details see Supplemental Table 3) and *bmp15-/-;inha-/-* (Fig. S5C, for details see Supplemental Table 4) compared to the control (*bmp15+/+;inha+/+*). We have also discussed this substantially in the Discussion part. We thank the Reviewer for bringing up this intriguing and interesting issue.

**Major point 2:**

Figure 9B: Authors show that *cyp19a1a* mRNA is increased by E2 treatment in the PG of *bmp15* mutants. E2 is produced in PV. It would be more appropriate to examine PV.

**RESPONSE**

This is a very good point. Fortunately, we also isolated the PV follicles and preserved their RNA when performing the experiment. As commented, we have analyzed the expression of *cyp19a1a* in these samples from PV follicles and the data are now shown in the new Fig. 7B. In addition, we have also expanded the sample size of the liver on the expression of *vtg1* and *vtg3* in the mutants after E2 and fadrozole treatments. The data quality has improved significantly, and we appreciate the comment by the Reviewer.

**Major point 3:**

The presentation of Figure 11A-D is confusing. One cannot tell which genes are up- or down-regulated from the colors. The scale bars representing mRNA levels may need to be normalized against those of the control fish and the fold change presented as a log2 scale. Which two groups of samples are compared to obtain the asterisk?

**RESPONSE**

We thank the Reviewer for the suggestion. We have changed the format of data presentation from heatmaps to graphs in Fig. 11 A-D (now Fig. 9A-D), all relative to the control fish (*bmp15+/-;inha+/-;inhbaa+/-*).

**Minor point 1:**

Line 295, please add back the parenthesis for “Fig6A”.

**RESPONSE**

We thank the Reviewer for spotting this error. We have added the parenthesis for “Fig. 6A” (now Fig. 4A).

**Minor point 2:**

The image of 50-dpf b*mp15-/-* mutant in Fig.2A is not representative. Fig. 2F shows that most gonads (5/7) already have PV.

**RESPONSE**

We thank the Reviewer for spotting this. We have replaced the image with a more representative one for *bmp15-/-* mutant at 50 dpf in Fig. 2A (now Fig. 1A), which contained PV follicles as shown in Fig. 2F (now Fig. 1F).

**Minor point 3:**

It will be better to present the X- and Y-axes of Figure 2C and 2D as the percentage of gonads with certain follicular stage versus age, then the temporal delay is more evident. How was the ratio of delayed puberty onset calculated? The method of calculation should be briefly described in the legend and in more detail in the M&M section. The scale bar should also be labeled in Fig. 2B.

**RESPONSE**

We thank the Reviewer for raising this interesting issue. Our previous study demonstrated that the initiation of puberty in female zebrafish was strongly correlated with body size rather than age (Chen and Ge, 2012). In this study, we defined the threshold of body size that would trigger puberty onset, *i.e.,* body length (BL) of 1.8 cm and body weight (BW) of 100 mg. This study has caught a lot of attention in the field and was featured in the article “Animals on the verge: What different species can teach us about human puberty” in Nature Medicine by S. Chakradhar (Nat Med 2018). In our aquatic system, this threshold is normally reached around 45 dpf.

In the present study, we presented the representative images of the control and mutant fish at different timepoints from 40 to 60 dpf in Fig. 2A (now Fig. 1A). To show the impact of *bmp15* mutation on puberty onset, we sampled three batches of fish (control and mutant) at 45 dpf (n=3; 18, 31 and 31 fish in each batch respectively) whose body size had reached the threshold and examined their ovaries by histology. The data shown in Fig. 2C (now Fig. 1C) are the percentages of fish in the three batches that had not shown puberty onset, which is characterized by the appearance of cortical vesicles in the leading oocytes. The female fish without PV follicles were considered to have delayed puberty onset. The ratio of delayed puberty onset was calculated as the number of females with delayed puberty onset divided by the total number of females. Fig. 2D (now Fig. 1D) shows the follicle stages of all fish examined from 40 to 60 dpf and their correlation with the body sizes (BL/BW). We have added the method of calculation in the figure legend.

As suggested, we have labeled the scale bar in Fig. 2B (now Fig. 1B).

**Minor point 4:**

Figure 4B: for easier understanding, the arrow indicating GP and BTs should be in different colors.

**RESPONSE**

As suggested, we have changed the color of arrows to dark blue for BTs.

**Minor point 5:**

Please describe the morphology of Fig. 10B in text L399.

**RESPONSE**

As suggested, we have added the description of Fig. 10B (now Fig. 8B) in the revised manuscript.

**Minor point 6:**

The abbreviations of EPN and LPN should be mentioned in Fig.13B legend.

**RESPONSE**

As suggested, we have added the abbreviations of EPN and LPN in the legend for Fig. 13B (now Fig. 11B.

**Minor point 7:**

Figure S1A: the meaning of black and white boxes should be described in figure legend.

**RESPONSE**

As suggested, we have added the meanings of black and white boxes to the Fig. S1 (now Fig. S2) legend.

**Reviewer #2:**

The manuscript by Zhai et al, investigates the relationship between BMP signaling and inhibin in oogenesis. The authors utilize elegant genetic experiments to demonstrate that BMP15 and activin/inhibin signaling control key events in oogenesis. Furthermore, they demonstrate that E2 production is a major output of these signaling pathways necessary for oogenesis as well as vitellogenesis production and uptake, which are likely downstream to E2. In addition, the authors compare the relative roles of GDF9 and BMP15 and, through double mutant analysis, show that they may have some antagonistic roles. Overall the experiments are thorough and support the conclusions made by the authors. This is a robust study and important body of work that enhances knowledge on signaling networks that are essential for oogenesis. The manuscript is clearly written and will be of broad interest to reproductive biologists and particularly fish reproductive biologists.

**RESPONSE**

We thank the Reviewer for the nice summary of our work and positive evaluation of our manuscript.

**Specific comment 1:**

Line 50: typo – “update” should be “uptake”.

**RESPONSE**

We thank the Reviewer for spotting the error. We have corrected it.

**Specific comment 2:**

Lines 255 – 263: It would be very interesting to follow the progression of sex reversal in the same fish using external landmarks (GP and BTs).

**RESPONSE**

We agree with the Reviewer that it would be interesting to follow the process of sex reversal in the same fish. However, to monitor the progression of sex reversal in the same fish would mean that we would not be able to examine gonadal changes at the same time points. Indeed, we performed such experiment in a recent study on EGF receptor knockout to monitor the process of sex reversal in the same fish (from 6 to 13 mpf) (Song et al. 2021).

**Specific comment 3:**

In the RT-PCR experiments shown in figure 1A, what stage/age were oocytes/ovaries that RNA was isolated from?

**Response**

To examine the spatial distribution of gene expression in the two cell types of follicles (oocyte and follicle layer), we separated the somatic follicle layer from the oocyte of FG stage isolated from adult female zebrafish (5 mpf) according to our previous reports (Ai et al. 2021). Total RNA was then isolated from denuded oocytes, follicle layers, and intact follicles, respectively. We have added this information in the manuscript.

**Specific comment 4:**

Line 378: there were not significantly different changes in gene expression between *bmp15* mutant and *bmp15; inha* double mutants. Treatment with E2 and fadrozole also did not show a statistically significant change in vtg3 expression.

**RESPONSE**

We thank the Reviewer for the comment. We have analyzed more samples during the revision and expanded the sample size of the liver. This has significantly improved the data quality with enhanced statistical power. The new results are now shown in Fig. 7C of the revised manuscript. The new data showed that the expression of *vtg1* and *vtg3* in the liver was significantly lower in *bmp15*-/- fish compared with control but increased significantly after E2 treatment. The expression also increased in the double mutant (*bmp15-/-;inha-/-*), but the increase was abolished by fadrozole treatment.

**Specific comment 5:**

Lines 88 – 91: Was fertility tested in the bmp15 mutant males?

**RESPONSE**

Disruption of *bmp15* did not cause any obvious abnormalities in testis development and spermatogenesis (Fig. S7). The single mutant males (*bmp15-/-* ♂) were fertile when crossed with WT females, and we have added photos showing male fertility in the new Fig. S7C.

**Specific comment 6:**

Figure S1: Panel C is missing.

**RESPONSE**

We thank the Reviewer for spotting the error. We have added it in Fig. S1 (now Fig. S2).

**Reviewer #3:**

Review of Rescue of BMP15 (*bmp15*) Deficiency by Mutation of Inhibin a (*inha*) Provides Novel Clues to How Bmp15 Controls Zebrafish Folliculogenesis.

General comments: To understand the roles of the oocyte signaling molecule Bmp15, Zhai et al. made a new allele of the zebrafish bmp15 gene, studied its phenotype, and then advanced understanding by making double mutants with inhibin- and activin-encoding genes and the related *gdf9* gene. In addition, they treated zebrafish with estrogen and an aromatase inhibitor to knockdown estrogen levels and examined the levels of vitellogenin yolk proteins in the blood. Results show that Bmp15 exerts its effect to allow vitellogenesis by acting through the activin/inhibin system mediated by estrogen. The results provide the fullest understanding of Bmp15 action in the development of ovarian follicles for any vertebrate.

One difficulty with interpretation is that to be able to interpret the triple mutant result, we need to know the phenotype of the *bmp15*; *inhbaa* double mutant and the *inha*; *inhbaa* double mutant. These animals would have come out of the crosses performed to obtain the triple mutant. Otherwise, the experiments are extensive, well described and documented, and provide a nice advance.

The data tables for the RNA-seq results should be included in the supplementary data because the text gives the impression that the up- and down-regulated genes highlighted were selected to further the authors’ interpretations. The text gave no data on how up- or down-regulated those mentioned genes were with respect to other genes some other researchers might have decided to prioritize.

**RESPONSE**

We thank the Reviewer for the nice summary of our work and the critical comment on our manuscript. As commented, we have added descriptions on the double mutants of *bmp15*;*inhbaa* and *inha*;*inhbaa* in the revised manuscript. This is indeed a very good point. Please refer to our response to Comment 26 below for details.

Also as suggested, we have added the RNA-seq data to the supplementary data (Supplemental Table 2-4). All the original RNA-seq data have been deposited at NCBI under the BioProject No. PRJNA849009.

**Specific comment 1:**

110 oocyte maturation whereas treatment with recombinant human BMP15 suppressed human chorionic gonadotropin (hCG). Does this imply that zebrafish has a human protein, or does it refer to some undescribed transgenic zebrafish, or to the zebrafish ortholog?

**RESPONSE**

This passage refers to early studies on BMP15 in zebrafish, conducted by Clelland et al. in 2006, 2007, and Tan et al. in 2009. These studies aimed to investigate the bioactivities of BMP15 in the zebrafish ovary using an in vitro approach after cloning the cDNA of the zebrafish ortholog of BMP15. As recombinant zebrafish BMP15 protein was not available, the authors used recombinant human BMP15 and human chorionic gonadotropin (hCG) to examine their actions and interactions on oocyte maturation in vitro. Unfortunately, since *bmp15* mutant females were unable to progress beyond the PV stage, we were unable to examine the impact of *bmp15* mutation on final oocyte maturation in the current study.

**Specific comment 2:**

133 suggest that both Gdf9 and Bmp15 are [both] critical.

**RESPONSE**

Corrected.

**Specific comment 3:**

136 activation or puberty onset (PG-PV transition). Define ‘puberty’ for a fish.

**RESPONSE**

Puberty is a crucial stage in the life cycle of vertebrates, marking the transitions from sexual immaturity to maturity. The definition of puberty onset varies across animal models, with different markers used. In zebrafish, the appearance of the first cohort of PV follicles in the ovary is used as a key indicator of puberty onset. Our laboratory was among the first to define female puberty onset in teleosts (Chen and Ge, 2013), and our research has attracted significant attention in the field. In fact, it was featured in the article "Animals on the verge: What different species can teach us about human puberty" in Nature Medicine by S. Chakradhar (Nat Med 2018).

As suggested, we have added this in the manuscript.

**Specific comment 4:**

151 Both gdf9 and bmp15 were detected exclusively in the denuded oocytes. According to zebrafish nomenclature rules, this sentence says that the gdf9 gene was only in oocytes. But the zebrafish gene is not only in oocytes but also is in follicle cells, it’s just not expressed in follicle cells. The sentence should say: ‘Both gdf9 and bmp15 expression was detected exclusively in the denuded oocytes.’

See: <https://zfin.atlassian.net/wiki/spaces/general/pages/1818394635/ZFIN+Zebrafish+Nomenclature+Conventions>.

**RESPONSE**

This is a very good point, and we thank the Reviewer for the suggestion. We have modified the sentence as follows: “Both *gdf9* and *bmp15* mRNAs were detected”.

**Specific comment 5:**

174 deletion was established that introduced an early terminator, [predicted to result] in synthesis … Unless the protein was isolated and shown experimentally to be a prematurely short protein, then this is only a prediction.

175 The loss of bmp15 was also. The bmp15 gene was not lost. A few nucleotides were lost and its mRNA was depleted and its function was lost.

**RESPONSE**

This is a very good point. As commented by the Reviewer, we have revised the descriptions at both places to be more precise.

**Specific comment 6:**

180 it is difficult to distinguish the homozygous mutant (*bmp15–/–*) from WT (*bmp15+/+*). It’s not so difficult to detect the homozygous mutant: the bmp15 mutant ovary eventually becomes a testis. I think the authors mean here that the homozygous mutation is difficult to distinguish from the wild type gene. Or the mutant allele is difficult to distinguish from the wild-type allele. A mutant is an animal. A mutation is a change in DNA sequence.

**RESPONSE**

We thank the Reviewer for the very good point. We have modified it.

**Specific comment 7:**

203 crossed the thresholds (1.80 cm [or] 100 mg). Just so it’s not a mathematical format for division.

**RESPONSE**

We thank the Reviewer for the comment and suggestion. We have changed “1.80 cm/100 mg” to “1.80 cm and/or 100 mg”, and we have also made the same change in other places.

**Specific comment 8:**

206 control group (*bmp15+/-*) Did the control group only have heterozygotes? Or did it have 2/3 heterozygotes and 1/3 homozygous wild types?

**RESPONSE**

The control group only had heterozygotes. In most experiments, we used heterozygote as the control because we did not observe any obvious difference between the wild type (+/+) and heterozygote (+/-) (Fig. S3) and it is easier to obtain heterozygous fish due to its high percentage in the offspring.

**Specific comment 9:**

207 BW and BL; however, about 40% mutants (bmp15-/-) Give actual numbers of fish as well as the %.

**RESPONSE**

We thank the Reviewer for the suggestion, and we have provided actual numbers (33/80) in addition to %.

**Specific comment 10:**

209 In addition, the mutant fish that [had] initiated puberty onset.

**RESPONSE**

Corrected as suggested.

**Specific comment 11:**

212 The use of the ‘puberty onset’ concept doesn’t quite feel right to me. I’m not for sure set against it, but I don’t see what’s gained by it and think that the follicle growth dynamics vs. time or vs. weight and length is what’s important.

**RESPONSE**

We thank the Reviewer for raising this important and interesting issue. As responded above to Comment 3, we were among the first to define puberty onset in teleosts (Chen and Ge, 2013). Basically, we used the formation of cortical alveoli in the first cohort of developing follicles as the marker to define female puberty onset. This occurs when the first wave of follicles develops from PG to PV stage. Furthermore, we have demonstrated that the puberty onset in female zebrafish is closely associated with body size (BL and BW) but not age (Chen and Ge, 2013), which is similar to the situation in mammals including humans. Because of this, it is not reliable to use time or age to evaluate puberty onset. This phenomenon has been fully confirmed by a series of studies in our laboratory (Chen and Ge, 2013; Lu et al., 2020; He et al., 2022; Zhao et al., 2022).

**Specific comment 12:**

Fig. 2. C and D are a bit difficult to compare. In some ways, it would be nice to have the two genotypes displayed on the same graph. That has the advantage of first, making the comparison easier to see, even though the dotted lines do help, and second, of visualizing better whether the weight to length ratios of the two genotypes are the same. It has the disadvantage of a confusing mix of colors with so many points even if the two genotypes were shown in different shaped symbols. The other variable is time, and it would be nice to see the length and/or weight of both vs time to see if the mutation affects growth rate generally or if it is specific for the follicles.

**RESPONSE**

We thank the Reviewer for the valuable suggestion. We have combined the data to display them in the same graph in Fig. 2D (now Fig. 1D). It does make the comparison easier to see. Fig. 2A (now Fig. 1A) shows changes of body size and follicle growth over time, respectively.

**Specific comment 13:**

216 histological examinations of *bmp15* mutants at sexual maturation stage (120 dpf). Should the text also give weight and length for this stage?

**RESPONSE**

We thank the Reviewer for the suggestion. We have added the BL and BW of the representative fish (control and mutant) at 120 dpf in Fig. 3 (now Fig. S3).

**Specific comment 14:**

217 control fish (bmp15+/+ and bmp15+/-) showed Here, both phenotypically wild type genotypes are listed, but in other places, only heterozygotes. Were the controls really different in these instances? Or does the text not accurately describe control genotypes?

217 bmp15+/+ and bmp15+/-) showed normal folliculogenesis with full range of follicles from PG to FG. The Fig 3A homozygous wt image looks to have more advanced follicles than the heterozygous ovary. Does the mutation have a partially dominant effect?

**RESPONSE**

We performed histological examinations of WT (+/+), *bmp15* heterozygotes (+/-) and *bmp15* homozygous mutant (-/-) at the sexual maturation stage (120 dpf). According to our observations, there is no significant difference in folliculogenesis of WT and heterozygous females (Fig. S3). The difference noticed by the Reviewer between the representative images (+/+ and +/-) was indeed within the normal range of individual variation. To avoid confusion, we have changed the representative image of *bmp15+/-* in Fig. 3A (now Fig. S3A) with another one, which is more comparable to that of the WT (+/+). Because the WT and heterozygote showed no visible difference, we chose to use the heterozygote as the control in all subsequent analyses because it is easier to obtain this genotype due to its higher percentage in the offspring.

**Specific comment 15:**

230 During this period, the testis and spermatogenesis were normal in mutant males. Some reviewers might insist on documentation and quantification for this claim, but I don’t think it’s necessary.

**RESPONSE**

We thank the Reviewer for the point. Despite that spermatogenesis is not our focus in the study, we have quantified the spermatozoa-filled luminal spaces in the testis of mutant males and compared them with the control. As shown in Fig. S7B, there was no difference between mutant testis (*bmp15-/-*, *bmp15-/-;inha-/-,* and *bmp15-/-;inha-/-;inhbaa-/-*) and control (*bmp15+/-;inha+/–;inhbaa+/-*).

**Specific comment 16:**

233 ovaries (8/21) and testes (10/21), The writing says there were 8 of 21 ovaries and 10 of 21 testes. Did one of the presumably 11 fish examined have only one ovary so that there were 21 instead of 22 ovaries? Or does the sentence mean ovaries in 8 of 21 fish examined, etc.?

**RESPONSE**

We are sorry for the confusion. The numbers in the brackets refer to numbers of fish examined, not numbers of ovaries or testes. To avoid confusion, we have changed the expression as follows: In addition to females (8/21) with ovaries and males (10/21) with testes, we also observed some mutant fish (3/21) with ovotestis, indicating sex reversal from females to males.

**Specific comment 17:**

235 (11/19) with [some other] fish at transitional state

**RESPONSE**

Changed as suggested.

**Specific comment 18:**

241 with a slim body shape, and they develop unique breeding tubercles (BT) on the pectoral fins [60]. Add here that the BT depend on testosterone.

**RESPONSE**

We thank the Reviewer for the suggestion. The generation of BTs in zebrafish has been shown to require androgens (Yu et al. 2018). We have added this point with reference.

**Specific comment 19:**

263 occurred asynchronously starting Change to: occurred at different times in different individuals.

**RESPONSE**

Changed as suggested.

**Specific comment 20:**

275 cortical alveoli but not yolk granules in the oocytes, the double mutant bmp15-/-;inha-/- showed normal vitellogenic growth beyond PV stage with large amount of yolk accumulated in the oocytes this is an interesting and important observation

**RESPONSE**

We thank the Reviewer for the supportive comment.

**Specific comment 21:**

272 mutant bmp15-/-;inha-/- and examined its folliculogenesis The text here must tell the reader what the phenotype is of the inha mutant compared to the bmp15 mutant before going into the phenotype of the double mutant in line 306.

**RESPONSE**

We thank the Reviewer for the suggestion. As suggested, we have modified the sentence as follows in the manuscript: “According to our report, the *inha-/-* single mutant displayed precocious puberty onset or early PG-PV transition, but with normal follicle growth; however, its oocytes failed to mature and ovulate, leading to female infertility (63)”.

**Specific comment 22:**

284 Transcriptome analysis of bmp15-/-, inha-/- and bmp15-/-;inha-/- mutant follicles In principle, the best experimental design would be to do transcriptomics also on the triple mutants and the inhbaa mutants and the inha;inhbaa double mutants, but for this work, I don’t think that’s necessary. The knowledge to be gained would be small compared to the expense and effort and should not hold up the publication of this work.

**RESPONSE**

We thank the Reviewer for the great suggestion. Indeed, a new PhD student is now continuing this line of work on BMP15, and the experiments suggested by the Reviewer will be considered in her project.

**Specific comment 23:**

301 expression patterns suggested that during folliculogenesis, Bmp15 may act as a facilitator [of what?], while inhibin acts as a depressant [of what? Generalized transcription? Text needs to specify this.]

**RESPONSE**

We thank the Reviewer for the suggestion. We have changed the sentence as follows: “Such expression patterns suggested that Bmp15 may act as a facilitator of folliculogenesis, while inhibin acts as a depressant.”

**Specific comment 24:**

351 feeding at 200 μg/g and water-borne exposure at 10 nM suppressed ovarian Provide here a brief interpretation phrase. Were the higher doses simply generally toxic for some reason? Also, it is important to know the effect of the estrogen treatments on the genital papillae of females and sex tubercles of males. Were the higher doses of estrogen inhibitory to only the ovary or to secondary sex characteristics as well?

**RESPONSE**

The Reviewer is correct. High doses of estrogens do have toxic effects on ovarian development, and we have reported this in our previous study (Chen et al. 2017). This study confirmed such effect. As suggested by the Reviewer, we have modified the sentence as follows: “Both feeding at 200 μg/g and water-borne exposure at 10 nM suppressed ovarian development, in agreement with our previous report on the toxic effect of E2 on folliculogenesis at high dosage [66]”.

Unfortunately, since we focused our attention on ovarian development and folliculogenesis, we did not explore the effects of E2 treatment on the secondary sex characteristics during this process. However, we agree with the Reviewer that this would be an interesting issue to explore in future studies.

**Specific comment 25:**

392 members. To test this hypothesis, we created a triple mutant using an inhbaa mutant
Text needs to tell reader the phenotype of the inhbaa single mutant.

**RESPONSE**

We thank the Reviewer for the suggestion. As suggested, we have added the following description on key phenotypes of the *inhbaa* single mutant: “The *inhbaa-/-* single mutant showed progressive deterioration of the ovary with reduced fecundity after 6 mpf, and its folliculogenesis stopped completely with follicles arrested at PG stage after18 mpf (67).”

**Specific comment 26:**

392 To be able to interpret the triple mutant result, we need to know the phenotype of the *bmp15;inhbaa* double mutant and the *inha;inhbaa* double mutant. These animals would have come out of the crosses performed to obtain the triple mutant.

**RESPONSE**

We thank the Reviewer for the insightful comment and suggestion. As the Reviewer mentioned, we did collect samples for the double mutants *bmp15-/-;inhbaa-/-* and *inha-/-;inhbaa-/-* from the crossing, and we have performed histology on these samples and added new figures in the revised manuscript (Fig. S6). Both double mutants showed similar phenotypes to that of *bmp15-/-* and *inha-/-* single mutants respectively, i.e., arrest at PV stage for *bmp15-/-;inhbaa-/-* and normal vitellogenic growth for *inha-/-;inhbaa-/-* (Fig. S6). These results are expected. First, the *bmp15* mutation caused a significant reduction of *inhbaa* expression at PV stage; therefore, a complete loss of *inhbaa* may not generate additional phenotype. As for *inha* and *inhbaa,* we recently proposed that among the three beta subunits of inhibin/activin (*inhbaa*, *inhbab* and *inhbb*), *inhbaa* was likely the major form that contributed to the formation of inhibin (Inha;Inhbaa) (Zhao, et al. 2022b). Based on this, the simultaneous loss of *inha* and *inhbaa* would produce similar phenotype to that of *inha* single mutant (Lu, et al. 2020a). However, if the loss of *inhbaa* also results in the loss of inhibin, one would expect that the double mutant of *bmp15-/-;inhbaa-/-* should also rescue the phenotype of *bmp15-/-.* The fact that *inhbaa-/-* could not phenocopy *inha*-/- in rescuing *bmp15-/-* provides an additional support to the idea we proposed that it was the increased expression of *inhbaa* that played a critical role in rescuing *bmp15-/-* by *inha-/-*. This provides further support to the mechanism we proposed for Bmp15 actions, and we sincerely thank the Reviewer for raising the issue.

We have added the new results and discussed them in the revised manuscript as follows: “The role of activin in rescuing the *bmp15-/-* phenotype by *inha-/-* is further supported by the observation that, despite being the major form of the β subunit that forms inhibin in zebrafish [67], the loss of inhbaa was unable to rescue *bmp15-/-* deficiency in the double mutant *bmp15-/-;inhbaa-/-*, unlike *bmp15-/-;inha-/-*. This finding strongly supports our hypothesis that the increased production and activity of activin from inhbaa in *bmp15-/-;inha-/-* is likely one of the main underlying factors responsible for the rescue of bmp15-/- phenotypes by *inha-/-*. In comparison to *bmp15-/-;inha-/-* mutant, which lack inhibin but have elevated activin derived from Inhbaa, the double mutant *bmp15-/-;inhbaa-/-* would have lost both inhibin and activin Aa.”.

**Specific comment 27:**

Fig. 10. I like the way that the red font makes it easier to tell which genes are homozygous for the mutation. A simple way to make it easier for the reader.

**RESPONSE**

We thank the Reviewer for the supportive comment.

**Specific comment 28:**

412 the femaleness was maintained, and sex change did not occur in both double and triple mutants That’s an interesting and important observation. A single sentence here interpreting that finding would be helpful.

**RESPONSE**

We thank the Reviewer for the suggestion. The follicles could grow to MV stage (~450 µm) in the double mutant (*bmp15-/-*;*inha*-/-) and nearly the size of EV stage (< 350 µm) in the triple mutant (*bmp15-/-;inha-/-;inhbaa-/-*). It seems that when follicles develop beyond the PV stage, they are less prone to deterioration and sex reversal. We have added the following sentence in the manuscript: “sex change did not occur in both double and triple mutants probably because the follicles become less prone to deterioration and sex reversal after PV stage”.

**Specific comment 29:**

443 Since Vtg production in the liver is tightly controlled by estrogens from the ovary,
Document for zebrafish by citation.

**RESPONSE**

As suggested, we have added one reference on E2 regulation of vitellogenin biosynthesis in the zebrafish liver (Levi et al. 2009) (68).

**Specific comment 30:**

Fig. 11. Scale bars should have units in the figure.

**RESPONSE**

As commented by another reviewer, we have changed the method of data presentation from heatmaps to graphs in the new Fig. 11 (now Fig. 9).

**Specific comment 31:**

452 At the pituitary level, *lhb* showed no response. The pituitary isn’t mentioned in the materials and methods, which should be fixed.

**RESPONSE**

We thank the Reviewer for the suggestion. To quantify the levels of gonadotropin expression in each individual pituitary, the gland from each fish was removed immediately following decapitation for total RNA extraction and real-time qPCR analysis according to our previous report (Chen and Ge 2012). As suggested, we have added the details in the Materials and Methods.

**Specific comment 32:**

460 signal for fshb in the triple mutant (Fig. 11E). It would be helpful to have a phrase here that interprets this result in terms of feedback signaling between gonad and pituitary.

**RESPONSE**

This is a very good point, and we thank the Reviewer for the suggestion. Similar to the situation in mammals, pituitary FSH is subject to negative feedback regulation by both inhibin and steroids from the gonads. As expected, the loss of inhibin caused an increase of *fshb* expression in the pituitary as we reported recently (Lu et al. 2020) due to the loss of inhibition by inhibin. A further increase in *fshb* expression in the triple knockout (*bmp15-/-;inha-/-;inhbaa-/-*) was likely due to the reduced negative feedback by gonadal estrogens (Fig. 9E) in addition to the loss of inhibin. We have added the following in the manuscript: “The further increase in fshb expression in the triple mutant could be due to the loss of both inhibition by inhibin and reduced negative feedback by estrogens.”.

**Specific comment 33**

482 Follicle composition analysis showed [many fewer] vitellogenic follicles in the ovary.

**RESPONSE**

We thank the Reviewer for the suggestion. We have corrected it as suggested.

**Specific comment 34:**

485 and 55.0% respectively while the ratio dropped significantly to only 22.6% in the triple mutant (Fig. 12D). It would be helpful to have a brief phrase here to interpret this observation.

**RESPONSE**

We have added the following at the end of the sentence as suggested: “suggesting impaired vitellogenic growth of follicles in the triple knockout with the loss of *inhbaa*.”.

**Specific comment 35:**

490 observed either in the double mutant of bmp15 and inha. It would be helpful to remind reader of the spermatogenesis in inha and inhbaa single mutants here and to say in a phrase the significance.

**RESPONSE**

We have added the following in the sentence with references: “similar to our observations in *inha* and *inhbaa* single mutants [64, 65].”

**Specific comment 36:**

513 The phenotypes of the published bmp15 mutant from the draper lab are different from the phenotype reported here in that the other allele allowed females to develop.

**RESPONSE**

We are grateful to the Reviewer for highlighting the novel contributions of our study in comparison to the previous study by Dranow et al. (2016). Both studies have demonstrated the arrest of follicle development at the PV stage in the absence of Bmp15, indicating consistency between the findings. However, our study goes beyond this by providing additional evidence that Bmp15 also plays a role in promoting the PG-PV transition or puberty onset.

Furthermore, our study offers valuable insights into the mechanisms by which Bmp15 controls early follicle development. We accomplished this through combination with other allele mutations (*inha-/-*, *inhbaa-/-* and *gdf9-/-*), analysis of gene expression, and pharmacological approach. Together, these approaches have provided critical clues regarding the action mechanisms of Bmp15 in controlling early follicle development.

**Specific comment 37:**

572 The BTs seemed to be a sensitive marker for the onset of sex reversal as they appeared even before any testicular tissues were visible in the ovary. This is interesting because the BTs arise due to testosterone. I would conclude that the testosterone level must increase before morphologies change detectably.

**RESPONSE**

We agree with the Reviewer on androgen dependence of BT development. Indeed, this has been well documented in a previous study (Yu et al. 2018). We have added the following sentence in the manuscript: “The development of BTs has been shown to be androgen-dependent in zebrafish (62).”

**Specific comment 38:**

581 Our recent study showed that the loss of gdf9 gene in zebrafish. When the text uses the italicized gene name nomenclature, like gdf9 here, it means the gene, so the word ‘gene’ is not required after the gene name, here after gdf9.

**RESPONSE**

As commented, we have deleted the word “gene”.

**Specific comment 39:**

582 arrest of follicle development at the PG stage. Give citation.

**RESPONSE**

The reference has been added.

**Specific comment 40:**

642 Roles of [the] activin-inhibin system

**RESPONSE**

Revised as suggested.

**Specific comment 41:**

643 One of [the] interesting findings

**RESPONSE**

Revised as suggested.

**Specific comment 42:**

Fig. 13A. Nice summary figure. It would be good to put gdf9 on it as well as the various bmp15 variants. 746 The loss of gdf9 resulted in a complete arrest of follicle development at PG stage without formation of cortical alveoli Show in fig13a.

**RESPONSE**

We thank the Reviewer for the encouraging comment. We have added the phenotype of *gdf9* mutant female in Fig. 13A (now Fig. 11A) according to the suggestion by the Reviewer.

**Specific comment 43:**

Fig. 13B Many other mutants besides bmp15 and gdf9 result in oocytes dying and the juvenile ovary becoming a testis, including cyp19a1a, fancl, fancd1, foxl2a, foxl2b, igf3, and others. The position in 13B where some of these disrupt development should be indicated, how do these differ from the sex reversal in bmp15?

**RESPONSE**

We appreciate the Reviewer's comment regarding the involvement of various factors in controlling early oocyte/follicle development in zebrafish. In addition to the factors mentioned by the Reviewer, some other factors are also involved in the regulation of early follicle development including FSH receptor (*fshr*), EGF receptor (*egfra*), pituitary growth hormone (*gh1*), anti-Mullerian hormone (*amh*), and the type II receptors of bone morphogenetic proteins (*bmpr2a* and *bmpr2b*). The loss of these factors can also lead to an arrest of follicle development at the PG stage, often accompanied by sex reversal.

However, since our present study did not specifically investigate the mechanism of sex reversal induced by *bmp15* mutation, we are afraid that inclusion of these factors and discussing their differences from *bmp15* in terms of sex reversal would exceed the scope of this study. Indeed, we are now planning a review article on early folliculogenesis in teleosts, which will cover all the factors mentioned.

Therefore, we hope that we will be allowed to retain the original Fig. 13B (now Fig. 11B) to focus solely on *bmp15* and *gdf9*.

**Specific comment 44:**

763 (vitellogenin synthesis and update) while suppress ING the formation of cortical alveoli
advancement of some follicles into early PV stage (PV-I), somewhere between gdf9-/- 770 and bmp15-/-. It’s unclear what is between these two mutant genotypes.

**RESPONSE**

We have replaced “suppresses” with “suppressing” as suggested. The difference between of *gdf9-/-;bmp15-/-* and *gdf9-/-* in follicular development is very subtle. However, In contrast to *gdf9-/-*, which resulted in a complete halt of follicle development at the PG stage without cortical alveoli, some follicles in *gdf9-/-;bmp15-/-* could progress into the early PV stage (PV-I) with small cortical alveoli. This phenotype can be considered as an intermediary between the characteristics of *gdf9-/-* (complete arrest at PG stage) and *bmp15-/-* (fully developed PV stage). We have revised the sentence in the manuscript to make this clearer.

**Specific comment 45:**

841 in males [respectivley (this word isn’t necessary and is misspelled)]

**RESPONSE**

We thank the Reviewer for spotting this error. It has been corrected.

**Specific comment 46:**

848 Follicle staging and quantification. Tell how these stages correspond to the Selman stages (https://doi.org/10.1002/jmor.1052180209) used by most in the field to improve comparison with other work.

**RESPONSE**

We thank the Reviewer for the comment. In our studies, we classify follicles into six stages: PG, PV, EV, MV, LV, and FG. This staging system, gaining popularity in current literature, offers the advantage of naming each stage based on its distinct structural characteristics. To ensure comparability with Selman's staging system (Stage I-III), we have also provided the corresponding Selman's stage in the brackets after each stage.

**Specific comment 47:**

856 largest sections spaced at least 60 μm apart. Does that mean 3 sections that are each 12 sections (=60µm/5 µm sections) apart? Or the individual follicles measured on each of the 3 sections were all 60 µm away from any other follicle measured on that section?

**RESPONSE**

We measured follicle diameters on three sections spaced at least 60 µm apart, not follicles that were 60 µm away from other follicles on the same section. To make it clearer, we have modified the sentence as follows: “To quantify follicle composition in the ovary, we performed serial longitudinal sectioning of the whole fish at a thickness of 5 µm. We measured the follicle diameters on three sections that were spaced at least 60 µm apart using the NIS- Elements BR software (Nikon). Among the three sections, the middle one represented the largest in the series.”.

**Specific comment 48:**

861 The [phenotypic] sex of zebrafish was identified…

**RESPONSE**

Changed as suggested.

**Specific comment 49:**

871 Fertility assessment Need to tell the age of the fish tested.

**RESPONSE**

The age of fish used for fertility test was 4 mpf for both females and males. We have indicated this in the manuscript as suggested.

**Specific comment 50:**

877 per female within three hours after spawning and the number of [surviving] embryos was…How was living vs. dead determined at 24h? Maybe the embryo looked ok at 24h, but was actually dead?

**RESPONSE**

We changed “survived” to “surviving” as suggested. We counted the total number of ovulated eggs three hours after spawning. These eggs included both living and dead ones since they were difficult to distinguish at this time. However, after 24 hours, the dead embryos became opaque, while the living ones remained transparent, enabling a clear differentiation between the two types. We have made minor changes in the sentence to make it clearer.

**Specific comment 51:**

912 medium (Sigma-Aldrich). We immediately dispersed the ovaries and isolated PG stage. For the method to be repeatable, the text has to tell how the ovaries were dispersed.

**Response**

We thank the Reviewer for the comment. We have revised the manuscript as follows: “All female fish were genotyped by HRMA, and their ovaries were carefully removed and placed in a microtube containing 1 mL L15 medium (Sigma-Aldrich). We used a BD Microlance 26G needle (BD, San Diego, CA) to remove fat tissues and ligaments surrounding the ovaries. The ovarian fragments were gently pipetted several times using a transfer pipette (JET Biofil, Guangzhou, China) to separate the follicles. The PG follicles were then isolated by passing the isolated follicles through a 150 μm filter (SPL Lifesciences, Waunakee, WI). The isolated PG follicles (< 150 μm) were collected by centrifugation at 3500 rpm (5 min) for transcriptome (RNA-seq) analysis.”.

**Specific comment 52:**

920 Cluster Profiler R package. The RNA-seq data are available at NCBI under the BioProject No. PRJNA849009. The authors must have the xl spreadsheet available as a supplemental table with this publication.

**RESPONSE**

We thank the Reviewer for the suggestion. The Excel spreadsheets of RNA-seq results are now provided in Supplemental Table 2-4.

**References**

Ai, N., L. Liu, E. S. Lau, A. C. Tse, and W. Ge. 2021. 'Separation of Oocyte and Follicle Layer for Gene Expression Analysis in Zebrafish', *Methods Mol Biol*, 2218: 1-9.

Chen, W., and W. Ge. 2012. 'Ontogenic expression profiles of gonadotropins (*fshb* and *lhb*) and growth hormone (*gh*) during sexual differentiation and puberty onset in female zebrafish', *Biology of Reproduction*, 86: 73.

Levi, L., I. Pekarski, E. Gutman, P. Fortina, T. Hyslop, J. Biran, B. Levavi-Sivan, and E. Lubzens. 2009. 'Revealing genes associated with vitellogenesis in the liver of the zebrafish (Danio rerio) by transcriptome profiling', *BMC Genomics*, 10: 141.

Lu, H., C. Zhao, B. Zhu, Z. Zhang, and W. Ge. 2020. 'Loss of inhibin advances follicle activation and female puberty onset but blocks oocyte maturation in zebrafish', *Endocrinology*, 161: 1-19.

Song, Y., W. Chen, B. Zhu, and W. Ge. 2021. 'Disruption of epidermal growth factor receptor but not EGF blocks follicle activation in zebrafish ovary', *Frontiers in Cell and Developmental Biology*, 9: 750888.

Yu, G., D. Zhang, W. Liu, J. Wang, X. Liu, C. Zhou, J. Gui, and W. Xiao. 2018. 'Zebrafish androgen receptor is required for spermatogenesis and maintenance of ovarian function', *Oncotarget*, 9: 24320-34.
