# Supplementary material for: Rescue of bmp15 deficiency in zebrafish by mutation of inha reveals mechanisms of BMP15 regulation of folliculogenesis
Source: PLoS Genet. 2023 Sep 15;19(9):e1010954. doi: 10.1371/journal.pgen.1010954 (PMC10529593; doi:10.1371/journal.pgen.1010954)
Supplement: Attachment — Submitted filename: Response to Reviewers comments.docx [file pgen.1010954.s013.docx]

**Response to Reviewers’ comments**

Once again, we would like to express our gratitude to the reviewers for their constructive and helpful comments, which have further improved the manuscript. We have revised the manuscript accordingly and our responses to reviewers’ comments are summarized below.

**Reviewer 1**

Authors have revised many parts of the manuscript and presented their novel findings more clearly. There are only a few minor points.

**RESPONSE:**

We thank the Reviewer for endorsing our revisions.

**Specific comment 1:**

Line 530: “…suggesting impaired vitellogenic growth of follicles in the triple knockout with the loss of inhbaa.” Authors may mean “inha” instead of “inhbaa”. The expression level of inhbaa in the triple mutant compared with other mutants or control are not shown in this manuscript.

**RESPONSE:**

We thank the Reviewer for spotting this error. We have revised the sentence to “…suggesting impaired vitellogenic growth of follicles in the triple knockout.”

**Specific comment 2:**

Line 1381:"the number in the box indicates the sample size.", The boxes are absent in Figure 2C. This sentence can be deleted.

**RESPONSE:**

We thank the Reviewer for spotting this. We have deleted the sentence as suggested.

**Specific comment 3:**

Figure 9C and D: The data and the statistical analysis are not mentioned in the text. It will be nice to mention the statistical analysis for the data for esr2a, esr2b, fshb and lhb, even when they are not statistically significant.

**RESPONSE:**

We thank the Reviewer for the valuable suggestion. As suggested, we have added description of statistical analysis in the text for *esr2a, esr2b, fshr and lhb* (Fig. 9A, C and D).

**Reviewer 2**

The authors have done a nice job addressing my previous comments.

**RESPONSE:**

We thank the Reviewer for endorsing our revisions.

**Reviewer 3**

Rereview Zhai Rescue of bmp15 deficiency in zebrafish by mutation of inha reveals mechanisms of BMP15 regulation of folliculogenesis. In general, the authors’ responses to the critiques are acceptable. They didn’t always do what the reviewers asked, but either responded in some other acceptable way or argued appropriately for another solution. In making the revision, a number of other generally minor problems arose, as detailed below, which should be fixed.

**RESPONSE:**

We thank the Reviewer for endorsing our revisions and constructive comments, which have helped to further improve the quality of our manuscript.

**Specific comment 1:**

33 Interestingly, the blockade of folliculogenesis and sex reversal in bmp15 mutantS could. In addition, saying that the blockade is rescued makes it seem that oogenesis is normal in the double mutant. Saying ‘partially rescued’ would correspond better to the results.

**RESPONSE:**

This is a very good point and we thank the Reviewer for the suggestion. We have added “partially” to make the description more accurate throughout the manuscript.

**Specific comment 2:**

53 Bone morphogenetic protein 15 (BMP15) is a protein produced by egg cells in animals. Not all animals.

**RESPONSE:**

We thank the Reviewer for the point. We have changed this sentence to “Bone morphogenetic protein 15 (BMP15) is an oocyte-secreted growth factor that plays an essential role in female reproduction.”.

**Specific comment 3:**

69 a developing oocyte and surrounding somatic follicle cells (granulosa and theca cells) Theca cells are not generally considered to be follicle cells.

**RESPONSE:**

As commented by the Reviewer, we have changed this sentence to “follicles in vertebrates consist of a developing oocyte and surrounding granulosa and theca cells”.

**Specific comment 4:**

127 flounder ovarian cell line suppressed expression of steroidogenic genes including aromatase (cyp19a1a), in contrast to gdf9 [41]. It’s unclear whether gdf9 acts different than bmp15 or cyp19a1a in this sentence construction.

**RESPONSE:**

We thank the Reviewer for the comment. Overexpression of Bmp15 and Gdf9 in the flounder ovarian cell line resulted in suppression and stimulation of steroidogenic enzymes respectively. To avoid confusion, we have removed “in contrast to *gdf9*” in the sentence. The contrasting effects of Bmp15 and Gdf9 were discussed in the Discussion part.

**Specific comment 5:**

133 Disruption of bmp15 gene in zebrafish resulted in follicle blockade at previtellogenic (PV) stage followed by sex reversal from females to males. Give citation.

**RESPONSE:**

We have added the citation as suggested.

**Specific comment 6:**

145 mutant (bmp15-/-) were arrested at later stage. Interestingly, lrp1ab, lrp5 and lrp6 175 were all expressed in both follicle cells and oocytes whereas lrp2a was exclusively expressed in the follicle cells (Fig. S1A). What does that finding say about the likelihood these proteins are actual Vtg receptors?

**RESPONSE:**

This is a very good point and we also thought deeply about it while drafting the manuscript.

As mentioned in the manuscript, the first putative Vtg receptor in fish was identified in the rainbow trout by homology-based PCR cloning and demonstrated to belong to the LRP subfamily of the LDLR superfamily. While the Vtg receptor has not been characterized in zebrafish, studies in other fish species suggest that it may likely be a member of the LRP subfamily.

The temporal expression profiles of *lrp1ab*, *lrp2a*, *lrp5* and *lrp6* (members of LRP family) showed a strong correlation with vitellogenesis. However, as noted by the Reviewer, the spatial distribution of these proteins in the follicle does not appear to support their potential roles as Vtg receptors. Rather than being exclusively expressed in the oocyte as expected, these proteins were also expressed in the follicle cells. This raises two intriguing questions. First, are these LRP members involved in Vtg uptake as its potential receptor(s)? Second, what is the biological significance of their expression in the follicle cells? These would be interesting issues to explore in our future studies. Given the current uncertainties about the functional roles of *lrp1ab*, *lrp2a*, *lrp5* and *lrp6*, we have refrained from drawing definitive conclusions about their involvement in Vtg uptate. Nonetheless, as members of the LRP family, their significant increase in expression in both *inha-/-* single mutant and *bmp15-/-;inha-/-* double mutant suggests that the heightened expression of putative Vtg receptors might be one mechanism contributing to the rescue of *bmp15-/-* phenotype by *inha-/-*.

**Specific comment 7:**

212 cortical alveoli appear first as a single layer of small vesicles (PV-I; 143±2 μm. The sentence says that the vesicles are 143µm in diameter. I suspect that is incorrect. Say specifically it is the oocyte or the total follicle that you are measuring.

**RESPONSE:**

We thank the Reviewer for pointing out this. The measurements refer to the sizes of follicles. To make it clearer, we have revised sentence to “Upon entering the PV stage, the cortical alveoli appear first as a single layer of small vesicles in PV-I follicles (143±2 µm), then larger ones in PV-II follicles (180±11 µm) and finally multiple layers in PV-III follicles (254±22 µm)”.

**Specific comment 8:**

316 To understand how mutation of inha could PARTIALLY rescue the phenotypes of bmp15 mutant, we Please fix this throughout the text.

**RESPONSE:**

We thank the Reviewer for the valuable suggestion. We have made corrections throughout the manuscript as suggested.

**Specific comment 9:**

317 performed a transcriptome analysis on PG follicles from WT (bmp15+/+;inha+/+), Was RNA-seq performed on follicles only? Or on complete ovaries? If the former, then if you say ‘dissected follicles’, there’s no ambiguity.

**RESPONSE:**

The RNA-seq analysis was performed on follicles only. We have modified the sentence as follows: “we performed a transcriptome analysis on PG follicles isolated from WT…”.

**Specific comment 10:**

326 genotypes (bmp15-/-, inha-/- and bmp15-/-;inha-/-) and These two genotypes are identical, so that’s confusing.

**RESPONSE:**

We thank the Reviewer for the comment. The genotypes in the brackets refer to three genotypes rather than two (*bmp15-/-, inha-/-* and *bmp15-/-;inha-/-*). To make it clearer, we have revised it to (single mutants: *bmp15-/-* and *inha-/-*; and double mutant: *bmp15-/-;inha-/-*).

**Specific comment 11:**

326 e similarity between inha-/- and bmp15-/-;inha-/- It would be better, instead of just saying inha-/-, the text would say bmp15+/+;inha-/-. This would help throughout the text when there’s a mix of double and single mutants being discussed together.

**RESPONSE:**

We thank the Reviewer for the valuable suggestion. We have changed “*inha-/-*” to “*bmp15+/+;inha-/-*” and “*bmp15-/-*” to “*bmp15-/-;inha+/+*” to avoid confusion.

**Specific comment 12:**

331 marco, nr1d2a and lrp2a, which were down-regulated in bmp15-/- follicles but upregulated Marco is a macrophage gene. So if the follicles were dissected, these macrophages were apparently infiltrating the follicle. Does that deserve a comment?

**RESPONSE:**

We chose a set of genes, including *marco,* to illustrate the changes of expression patterns in different mutants. We refrained from delving into the biological implications of these changes, as such a discussion would exceed the scope of our current study.

**Specific comment 13:**

434 at PG stage after18 mpf [67]. Also, tell whether there was ovotestis, sex reversal.

**RESPONSE:**

We have added the following in the revised manuscript “Despite ovarian deterioration, the inhbaa-/- mutant was able to maintain its female characteristics without undergoing sexual reversal”.

**Specific comment 14:**

474 of the genes examined were significantly up-regulated in the ovary of inha-/- mutant Since follicles are being investigated, not whole ovaries, it’s probably better to say ‘follicles’ here than ‘ovary’, even though both are probably true.

**RESPONSE:**

As suggested by the Reviewer, we have changed “ovary” to “follicles” to make it clearer.
